# Advances in upper gastrointestinal endoscopy

**DOI:** 10.12688/f1000research.6961.1

**Published:** 2015-12-16

**Authors:** David G. Graham, Matthew R. Banks

**Affiliations:** 1 Department of Gastroenterology, University College London Hospital, London, UK

**Keywords:** gastrointestinal endoscopy, gastrointestinal tract, optical coherence tomography, confocal laser endomicroscopy, endoscopy, endomicroscopy

## Abstract

The rapidly moving technological advances in gastrointestinal endoscopy have enhanced an endoscopist’s ability to diagnose and treat lesions within the gastrointestinal tract. The improvement in image quality created by the advent of high-definition and magnification endoscopy, alongside image enhancement, produces images of superb quality and detail that empower the endoscopist to identify important lesions that have previously been undetectable. Additionally, we are now seeing technologies emerge, such as optical coherence tomography and confocal laser endomicroscopy, that allow the endoscopist to visualize individual cells on a microscopic level and provide a real time, *in vivo* histological assessment. Within this article we discuss these technologies, as well as some of the results from their early use in clinical studies.

## Introduction

Whilst the intricate system of mirrors and lens, with a lamp flame as a light source, that was the early endoscope provided a unique opportunity to visualize the gastrointestinal tract, it was not clinically practical until the flexibility of fiber-optics was introduced and a new era of endoscopy, pioneered by Curtiss and Hirschowitz, began ^1^. Further advances, in the development of a charge couple device that produced electronic images and linked the endoscope to the computer, prompted a surge in technological endoscopic advancements and revolutionized gastroenterology ^2^.

The ability to visualize and sample the gastrointestinal tract is now an accepted norm for gastroenterologists and, as such, the focus is now on enhancing the diagnostic yield further by enabling endoscopists to accurately diagnose microscopic pathology, particularly high-risk pre-malignant lesions or early stage cancers, where their detection would significantly alter the prognosis. Furthermore, the promising developments in optical biopsy techniques aim to address the issues of variability in histopathological assessment of biopsy samples and the ever-increasing demand on these services ^3^.

This article aims to explore some of the exciting advancements in endoscopic upper gastrointestinal imaging that are presently being used within healthcare, as well as those that are currently being developed which provide great potential.

## Advances in digital imaging

The well recognized rapid advances in television technology that provide the viewer with a more engaging, immersive experience have also occurred in endoscopic imaging. Endoscopists are becoming increasingly able to detect subtle, minute mucosal changes that were previously indistinguishable from normal tissue. This is due to improving standards in resolution and magnification, combined with image enhancement techniques available to the endoscopist at a touch of a button.

### Endoscopy in high definition and high magnification

Similar to the changes in television, the endoscope has switched from “standard definition” to digital, high-definition white light imaging. Three companies (Pentax, Olympus, and Fujinon) are presently leading the field in endoscopic imaging, and currently have advanced high-definition endoscopic systems available for clinical use. They offer the improved ability to distinguish subtle mucosal differences in tissue in close proximity to each other, which is partly due to improved resolution (defined by pixel density). Standard definition endoscopes offered images of approximately 300,000 pixels whereas the new high-definition endoscopes, when combined with the latest processors, can achieve image quality of over 2 million pixels. Furthermore, all three of these companies offer endoscopes with high magnification to further enhance imaging. A standard endoscope magnifies an image by 30–35 times normal. However, these companies produce high-definition endoscopes that can optically magnify images by up to 150 times (Pentax MagniView, Olympus near-focus imaging, and Fujinon optical magnification) ^4^.

### Image enhancement

In addition to high-definition white light imaging, current market-leading endoscopes also provide further image enhancement by offering the endoscopist the ability to filter certain wavelengths of light. iScan (Pentax), OE (Pentax), narrow-band imaging (NBI, Olympus) and Fuji Intelligent Chromo Endoscopy (FICE, Fujinon) are all post-processing optical technologies that are designed to enhance subtle architectural or vascular patterns on the mucosal surface, and therefore enhance an endoscopist’s ability to detect subtle lesions within the gastrointestinal tract. However, these techniques do have their limitations. For example, NBI is excellent at demonstrating the changes in microvasculature seen in early gastric and esophageal lesions. However, it is not so effective at identifying some of the associated mucosal changes seen in these conditions, an area in which FICE excels ^5^. Similarly, the viewing of lesions at a distance is problematic with these techniques ^5^. Blue laser imaging (BLI, Fujinon) has been developed to overcome these issues, through combining narrow-band laser light with high-definition white light.

**Figure 1.**
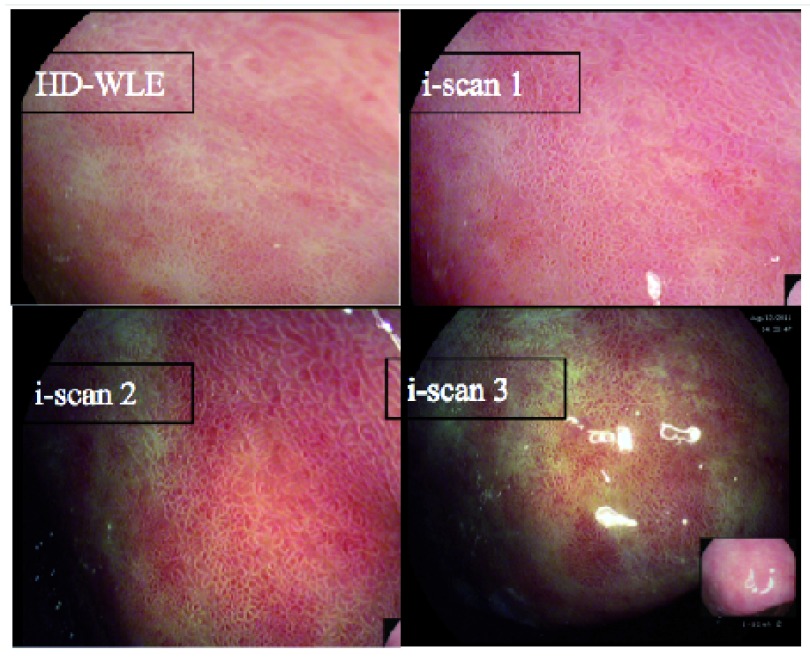
iScan (Pentax) images of Barrett’s esophagus demonstrating the mucosal surface and vasculature enhancement.

Whilst these techniques aim to offer an improved visualization of the upper gastrointestinal tract ( Figure 1), they still require the endoscopist to be suitably trained and engaged in utilizing them. There are no data on how often these techniques are actually applied in clinical practice, however, within our center we offer training for endoscopists from around the UK on these techniques and have anecdotally found their uptake is variable. Similarly, we have found the ability of these techniques to aid in detecting lesions varies according to the endoscopist’s interest. Our unpublished data found that specialist upper gastrointestinal endoscopists using iScan could detect dysplasia within a Barrett’s segment with 75% sensitivity. However, when general endoscopists were asked to detect dysplasia using iScan, this dropped to 55%. There are obviously other mitigating factors in this unpublished study, but this does demonstrate the need for these technologies to be combined with an endoscopist’s expertise and experience.

### High-definition, high-magnification endoscopes in practice

There have been many studies exploring the benefits of high-definition, high-magnification endoscopes. This work has mainly focused on Barrett’s esophagus, which is a pre-malignant condition of the esophagus. There is limited work investigating the benefits of these endoscopes in the stomach and small bowel. However, the work within the esophagus has shown these technologies to be useful. An example of this was demonstrated by Wolfsen *et al.*, focusing on dysplasia detection in Barrett’s esophagus (a high risk form of this disease which carries an increased cancer risk) ^6^. They compared high definition and NBI endoscopy (HD-NBI) with targeted biopsies to standard white light endoscopy and random biopsies. The study demonstrated that HD-NBI techniques provided a greater dysplasia yield (57% *vs* 43%) and required fewer biopsies ^6^.

Studies using these techniques in the stomach have had varying success. A study using iScan for the detection of pre-cancerous or early cancerous lesions within the stomach found that, although imaging quality was improved, there was little additional diagnostic benefit ^4^. However, recently Matsuo *et al.* found that using magnifying endoscopy with NBI (enhanced by the application of acetic acid) aided the diagnosis of early gastric cancers ^7^. Similarly, a study by Dohi *et al.* found that using BLI increased the detection of early gastric cancer with an accuracy, sensitivity, and specificity of 90.7, 84.6 and 92.4%, respectively, compared to 72.9, 30.8 and 84.8% using high-definition white light alone ^8^.

Work within the small bowel has also shown promise. Cammarota *et al.* performed a study of 191 patients that demonstrated that high definition magnification endoscopy had 95% sensitivity, 99% specificity, 95% positive predictive value, and 99% negative predictive value to detect the presence of any villous abnormality, and thus make a diagnosis within the small bowel without the need to biopsy ^9^.

### Molecular imaging

This is a rapidly growing discipline in medical imaging, utilizing unique molecular signatures for targeted imaging of pathology. This technique relies upon the development of exogenous molecular probes that specifically locate and highlight desired pathology. The potential for molecular imaging goes beyond that of just aiding in the detection of lesions. Other possible applications of this technology are within the field of therapy, where molecular imaging could enhance drug delivery and monitor drug response ^10, 11^.

**Figure 2.**
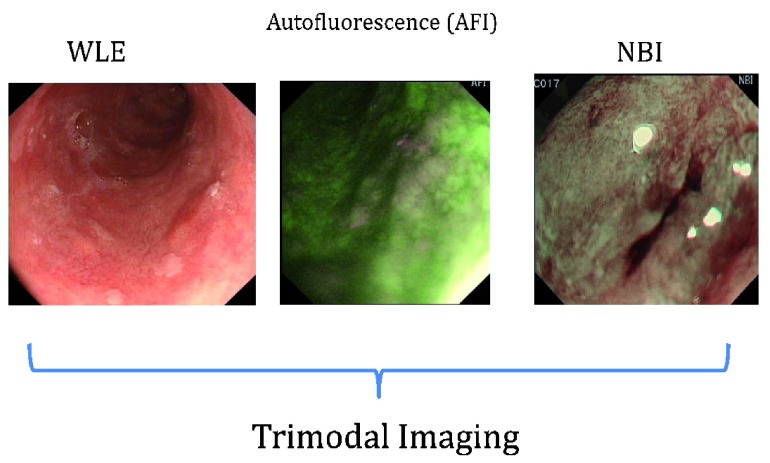
Trimodal endoscopic imaging of an early cancerous lesion in the esophagus.

Autofluorescence is an area of molecular imaging that is based upon the detection of natural tissue fluorescence emitted by endogenous molecules (fluorophores), such as collagen, flavins and porphyrins, producing a virtual chromoendoscopy technique ^12^. Studies demonstrated that dysplastic or cancerous tissue emitted a different autofluorescence spectrum compared to normal tissue. Consequently wide-field autofluorescence imaging was integrated with high-definition white light endoscopy and NBI to produce “trimodal endoscopic imaging” ^13, 14^. This technique is not yet in clinical use and there is little data from large-scale clinical trials, although the data from smaller studies appear promising ( Figure 2 below depicting trimodal imaging of an early cancerous lesion). Similarly, the use of near-infrared endoscopy and fluorescent activatable probes has limited clinical data, but early work demonstrates promise ^15^. Using autofluorescence, early esophageal squamous neoplasia was visualized more reliably than using white light endoscopy alone (79% *vs* 51%). Dysplasia within Barrett’s esophagus was detected more often using autofluorescence than white light endoscopy alone (90% *vs* 53%), but this was at the expense of a high false-positive rate of 81%. Finally the detection of early gastric cancers increased by 13% using autofluorescence, but again at the expense of a poor specificity ^12^.

## Optical biopsies

The need for effective optical biopsy techniques is apparent. As the field of endoscopy widens and lesions are more readily identified and removed, the workload for the already stretched histopathology and endoscopy departments increases. The cost of processing and analyzing tissue specimens removed endoscopically and then bringing the patients back for further follow-up procedures is spiraling. In addition to this, the existing random biopsy techniques utilized in areas such as Barrett’s esophagus surveillance are time-consuming, plagued with issues of missed cancers, and only sample around 5% of the mucosa ^16, 17^. It is clear that an effective “bed-side” immediate diagnostic technique would prove to be invaluable.

### Optical coherence tomography (OCT)

OCT has produced some promising data in the field of optical biopsy techniques. It relies upon the backscattering of light to provide cross-sectional images of tissue with high resolution and a scanning depth of 1–3 mm. The construction of an image through reflected light is similar to the use of acoustic waves in ultrasound, although, unlike ultrasound, neither a water or tissue apposition is required. However, the use of OCT in clinical practice has been limited due to imaging speed, and attempts to increase this have resulted in an unwanted loss of sensitivity ^18^. Optical Frequency Domain Imaging (OFDI) was developed to overcome the issue of image acquisition speed whilst maintaining sensitivity.

In 2013, an OCT system for esophageal imaging became commercially available (NvisionVLE, NinePoint Medical). This device uses a through-the-scope balloon that can acquire a 6 cm circumferential image in an automated scan. Clinical studies are underway exploring the device’s efficacy ^19^. Recently, Gora *et al.* published on their development of a tethered capsule providing OFDI imaging of the esophagus which potentially provides a simple, quick, and effective means for imaging the esophagus, however, clinical data are awaited ^20^.

The majority of published data focuses on esophageal pathology, as OCT images within the stomach are characterized by low tissue contrast and poor visualization. However, within the esophagus there have been some promising data. A study using 177 biopsy correlated OCT images in patients with Barrett’s esophagus found that this modality was capable of identifying dysplasia with a sensitivity of 83% and specificity of 75%. OCT has also been effectively used for the staging of esophageal tumors. In a study of 123 patients with esophageal squamous cell carcinomas, OCT correctly staged 95% of the lesions limited to the epithelium/lamina propria with higher accuracy than the existing endoscopic ultrasonographic technique ^21^.

### Confocal laser endomicroscopy (CLE)

This provides real-time histological assessment through high-resolution imaging, used as an adjunct to high-definition endoscopy. The key principal behind CLE is its ability to provide an in-focus image from a selected depth, whilst light from the out of focus planes are inefficiently collected. Unlike OCT, this technique relies upon fluorescent dye, most commonly injected fluorescein, prior to laser illumination. Whilst injection of fluorescein has an excellent safety profile, having been used extensively in ophthalmology, its use does add an additional process to the technique. However, this must be weighed up alongside the opportunity to obtain greater image contrast than in OCT ^22, 23^.

There are currently two CLE platforms; probe based (pCLE) and endoscope based (eCLE). The probe based system (Cellvizio confocal miniprobes, Mauna Kea Technologies, Paris, France) can only scan in a single plane, due to a fixed focal length, and provide an imaging depth between 40 and 70 mm depending on the probe used. For the upper gastrointestinal probe the maximal field view is 240 µm with a resolution of 1 μm. The AQ-Flex 19 Miniprobe has recently been developed for use through an EUS FNA (endoscopic ultrasound and fine needle aspiration) needle, however, there is little data on its clinical application ^23, 24^. The integration of a confocal microscope (Optiscan, Victoria, Australia) into an endoscope (Pentax, Tokyo, Japan) allowed for high-definition endoscopy to be performed simultaneously to eCLE with images displayed on dual monitors. eCLE provided a greater field view (475 mm) and was otherwise comparable to the performance of pCLE. The largest study comparing these two platforms in gastrointestinal disease found that pCLE offered shorter procedure times and comparable diagnostic yields to eCLE apart from in esophageal disease, where eCLE was found to offer better image quality and therefore improved examination ^25^.

**Figure 3.**
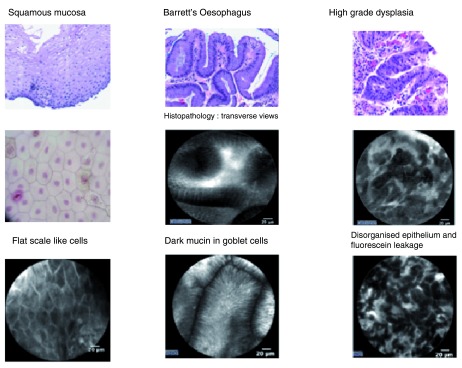
Confocal endomicroscopy (CLE) images demonstrating the ability to visualize individual cells and thus make an *in vivo* histological diagnosis. These images are taken from Barrett’s esophagus.

The clinical application of CLE in the upper gastrointestinal tract has provided promising results. Studies with the largest numbers have explored its use in detecting dysplasia within Barrett’s esophagus and gastric lesions, however, there are some data showing that CLE can be used to accurately diagnose Celiac disease ^26^. In Barrett’s esophagus, a recent randomized trial recruited 192 patients on routine Barrett’s esophagus surveillance, or those referred for confirmation of suspected dysplasia within their Barrett’s segment. This trial compared high definition endoscopy plus eCLE and targeted biopsy with high-definition endoscopy alone with random biopsies, and demonstrated that the use of CLE significantly improved the ability to detect dysplasia and early cancers within Barrett’s esophagus (34% *vs* 7%) whilst requiring fewer biopsies ^27^. The use of CLE would have eliminated the need for a biopsy in 65% of patients, which would have a potentially huge impact on healthcare resource provision ^27^. Similarly, CLE has been demonstrated to enhance diagnostic yield in pre-cancerous or early cancerous lesions of the stomach and can detect small intestinal pathology ( Figure 3), such as Celiac disease, with a 94% sensitivity and 92% specificity, which again would greatly reduce the burden of biopsies on the histopathology department ^23^. Due to the high cost of equipment, additional endoscopist expertise, and longer duration of the endoscopic procedure, CLE utilization was initially limited to enthusiasts and centers of excellence, but its use is now growing more widespread.

### Spectroscopy

The principles behind spectroscopy rely upon how objects of differing size and structure interact with light, typically light in the backward direction (towards the incident light). This provides insights into the molecular composition of the target tissue. Unlike other forms of optical biopsy techniques, the spectroscopic output is quantitative rather than being reliant upon user interpretation of an image, which potentially lends itself to inter-observer variation. Whilst this may be seen as an advantage to some, it requires a shift in the established approach to reaching a diagnosis, and consequently may be met with some skepticism ^16^.

Whilst there has been work looking at various properties of spectroscopic methods, an area that has a great deal of published data is that of inelastic (Raman) scattering spectroscopy. This measures signals obtained when the incident light undergoes wavelength shifts caused by energy transfer in the tissue. The different molecular composition and therefore molecular bonds of various tissue types (e.g., cancerous, dysplastic, normal) respond to energy uniquely and produce distinct shifts in light wavelength. *Ex vivo* studies assessing the ability of Raman spectroscopy to detect dysplasia in Barrett’s esophagus has shown sensitivities ranging from 73–100% and specificities of 90–100% ^19^, whereas a recent *in vivo* study demonstrated sensitivity of 87% and specificity of 84.7% ^28^. Within the stomach, Raman spectroscopy has demonstrated similar success in identifying gastric lesions with a sensitivity of 90% and specificity of 73.3% ^29^.

An important issue of these studies and an aspect of spectroscopy that has proven to be a stumbling block is that the majority rely upon a probe-based system. Accurately studying large areas of tissue with a probe is impractical in clinical practice and will likely continue to lead to missed lesions. Recent work addressing this issue has been published whereby an endoscopic polarized scanning spectroscopy system (EPSS) has been developed that is capable of performing rapid optical scanning and multispectral imaging of the entire esophageal surface and provide a real-time diagnosis. Early data on a small number of patients demonstrate a sensitivity of 92% and specificity of 96%, however, further studies with a larger cohort of patients are required ^30^.

## Summary

This article highlights just some of the many advancements in upper gastrointestinal endoscopy. As the science behind these technologies improves, so does the ability of the endoscopist to visualize and treat lesions within the gastrointestinal tract. It is without doubt that the improved ability to identify pre-cancerous or early cancerous lesions that have previously evaded even the most diligent endoscopist will have a significant impact on prognosis. Alongside this, the impact these technologies could have in reducing the need for biopsies and therefore the number of procedures will significantly alter the management of strained healthcare resources.

However, one must acknowledge that much of this technology is still in its early stages and is yet to be fully tested in clinical practice. Similarly, although these technologies provide an exciting platform for the endoscopist, one must remember that they are still reliant on the diligence and expertise of their user. These techniques are only as good as the quality of training and the acquisition of knowledge that precedes their use. We believe, however, that as with high-definition endoscopy, some of these technologies will emerge into mainstream use providing significant benefit to patient outcomes.
